# A Thematic Analysis of Career Adaptability in Retirees Who Return to Work

**DOI:** 10.3389/fpsyg.2016.00193

**Published:** 2016-02-17

**Authors:** Jennifer Luke, Peter McIlveen, Harsha N. Perera

**Affiliations:** ^1^School of Linguistics, Adult and Specialist Education, University of Southern QueenslandToowoomba, QLD, Australia; ^2^School of Education, University of New South WalesSydney, NSW, Australia

**Keywords:** retirement, bridge employment, career adaptability, career construction theory, career development

## Abstract

Retirement can no longer be conceptualized as disengagement, as the end of a person’s career, as it is in the life-span, life-space theory. Increasingly, retirees are returning to work, in paid, and unpaid positions, in a part-time or full-time capacity, as an act of re-engagement. Vocational psychology theories are yet to adequately conceptualize the phenomenon of retirees’ re-engagement in work. The research reported in this paper is the first attempt to understand re-engagement through the theoretical lens of career construction theory (CCT) and its central construct, career adaptability. The study involved intensive interviews with 22 retirees between the ages of 56 and 78 years (*M* = 68.24), who had retired no less than 1 year prior to the study. Participants were engaged in a discussion about their reasons for returning to the world of work. Thematic analysis of interview transcripts extracted evidence of the four career adaptability resources: concern, control, curiosity, and confidence. In addition, the influence of family and making a contribution were discerned as important themes. These findings are the first evidence that the CCT and career adaptability provide a new conceptual lens to theorize and conduct research into the phenomenon of retirement.

## Introduction

Retirement may be an existential transition that prompts many individuals to become temporarily introspective, giving pause to question past accomplishments and current life situation (Malette and Oliver, 2006). Psychological research into pre-retirement planning and post-retirement intentions and activity can make a positive contribution to policy and programs for older workers (Adams and Rau, 2011; Earl et al., 2015; Griffin, 2015), particularly the generation now exiting the workforce en masse: the Baby-Boomers (Quine and Carter, 2006; Shultz and Wang, 2011). It is against this backdrop—the putative exodus of skilled workers—that the notions of “encore career" (Figgis, 2012) and “bridge employment" (Wang and Shultz, 2010; Pleau and Shauman, 2013; Wang and Shi, 2014) became the focal points of the current research. Bridge employment is a substantial topic of research into retirement (Shultz and Wang, 2011; Beehr, 2014; Wang and Shi, 2014; Beehr and Bennett, 2015), particularly given that bridge employment may be beneficial to retirees’ health and well-being (Zhan et al., 2009) and organizations’ access to a flexible labor supply (Wang and Shi, 2014). The notion of bridge employment is usually associated with transition into retirement; it is not associated with transitioning out of retirement, back into the world of work. This ostensibly inverse phenomenon is under-theorized and there is a need to conceptualize re-engagement from a vocational psychological perspective.

The current research is a product of the zeitgeist to reform of career development—to make its ways of being, knowing, and doing, as a collection of disciplines and professional activities, relevant to a post-industrial world of work in which “decent work”, in context of the Sustainable Development Goals (United Nations, 2015), is posited as a human right for all (Blustein, 2006, 2013; Guichard, 2013). These scholars of decent work reflect on a world of work that is precarious and devoid of the benefits of traditional psychological contracts between employer and employee (e.g., mutuality, loyalty). The psychology of working framework (PWF; Blustein, 2006, 2013) posits three sets of fundamental needs that may be met by decent work: survival and power, social connection, and self-determination. With respect to older workers in need of decent work, one may ask, “What are the psychological dimensions of decent work for a retiree who returns to work?” PWF scholars (Sterns and Sterns, 2013) have identified issues associated with older workers (e.g., decline in physical and cognitive performance) and argued for workplace adjustments, including ergonomic enhancements to workspaces and equipment so as to prolong the worker’s productive years.

Toward the objective of conceptualizing retirees’ returning to work, the current research uses the career construction theory (CCT; Savickas, 2002, 2005, 2013) as an extension of the developmental life-span life-space theory (Super, 1980, 1990). A specific aim of the research is to explore the relevance of the CCT’s construct *career adaptability* to retirees’ experiences of working. Thus, the research explores the psychological factors that may contribute to an older person being actively productive in the workforce.

There are several general theories of adult development (Levinson et al., 1978; Erikson, 1993) and models of retirement (Shultz and Wang, 2011), including retirement as decision-making, an adjustment process, and a career stage (Wang and Shi, 2014). Notwithstanding their qualities, we focus on the vocational psychology perspective for two reasons. First, the volume of empirical research and professional applications of Super’s theory (Super, 1980, 1990) to working life and career is significant, extending over six decades (Hartung, 2013), and outweighs that of other theories, such as that of Levinson et al. (1978; Sharf, 2010). Second, from a developmental perspective on career, adjustment to career and working life is seen as central to and continuous with self-concept (Super, 1988, 1990).

The life-span, life-space theory comprises five stages (i.e., life-span) of growth, exploration, establishment, maintenance, and disengagement/decline, with each stage addressing career development tasks and roles (i.e., life-space). Retirement occurs in the final developmental stage of disengagement. Readiness and successful transition from one life stage to the next involves physical and psychological adjustment, representative of career maturity. Deceleration from employment and entry into retirement are activities included within the disengagement stage. Super acknowledged life events as a cause for a person to revisit earlier career life stages such as a retiree deciding to re-enter employment in a worker role. Accordingly, an individual who returns to work may be thought about as going through a process that Super described as a mini-cycling, whereby interests, abilities, and values are reappraised using processes seen in an earlier developmental stage such as the maxi-cycle stage, crystallization.

Recent research by Wang (2007) confirms the presence of three growth patterns in retirees’ transitions: the maintaining pattern (i.e., life satisfaction remains stable), recovering pattern (i.e., life satisfaction increases after leaving work), and U-curve pattern (i.e., life satisfaction initially dips after retirement and then recovers with an adjustment). Variations in these curves arise from “job characteristics (i.e., physical demands and work stress), job attitude (i.e., job satisfaction), health attributes (i.e., objective health declines), transition characteristics (i.e., retirement planning and timing), and family context variables (i.e., marital status, spouse working status, and marriage quality)” (Wang, 2007, p. 470). These patterns may be interpreted as variations of the stage disengagement and point to potential correlates of re-engagement in work.

The life-span, life-space theory does not, however, offer sufficient theoretical perspectives on the psychological processes associated with mini-cycling in post-retirement. Thus, the current research offers a conceptual and empirical response to the existential question posited in the broader literature of planning for retirement, that is, “What will I do?” (Adams and Rau, 2011). More specifically, the research addresses the question from the perspective of the CCT (Savickas, 2002, 2005, 2013): How does career adaptability manifest in individuals who return to work after several years of full-time retirement?

In formulating the research questions we attended to how CCT extends on the life-span, life-space theory (Savickas, 1997, 2002) and earlier renditions of the notions vocational maturity (Super, 1977), adjustment (Super, 1988), and adaptability (Super and Knasel, 1981), which evolved into CCT’s core construct, career adaptability (Savickas, 1997, 2005). Career adaptability comprises four resources: concern, control, curiosity, and confidence. Savickas (2013) summarized the qualities of these resources:

• Concern: planning, awareness, involvement, and preparation.
• Control: as decisiveness, assertiveness, discipline, and willfulness.
• Curiosity: inquisitiveness, exploration, risk taking, and an inquiring approach.
• Confidence: is expressed as efficacy, problem solving, and industriousness. (p. 158).

Recent research classified the behavior of retirees: continuers, adventurers, easy gliders, searchers, and retreaters (Maggiori et al., 2014). Continuers evince the maintaining pattern; they retain their occupational identity, may continue working at reduced hours and/or use their skills in a voluntary capacity. Adventurers enjoy the boon in time and use this resource to learn new skills and take on new projects. Easy gliders take a moratorium on commitments and leave all their options open. Searchers explore all that is available to them, try new opportunities, and frequently change their commitments. These three types may reflect Wang’s (2007) recovering or U-curve pattern. Adventurers, for example, may experience heightened life satisfaction in the joy of knowing what time is available to them to pursue rewarding activities; whereas searchers may experience a U-curve dip in satisfaction until they begin to choose and then engage in rewarding activities. Retreaters disengage and withdraw from life and could not be seen as adaptively adjusting to retirement in terms of Wang’s three patterns. Apart from retreaters, it is possible that all of these types may return to work but for very different reasons.

Subjective life expectancy (SLE) is a person’s estimate of the number of years of life he or she has remaining. Griffin (2015) suggests that an employee’s motivations turn toward social and emotional goals rather than learning and work goals, as a function of consciousness of years remaining and inevitable death. In a longitudinal study of older workers (*M* = 62 years), SLE not only predicted decision to retire, but also predicted return to work after retirement by those individuals who had already retired at Time 1 (Griffin et al., 2012). These findings suggested that a greater SLE is positively associated with return to work. Thus, the existential question “What will I do?” in retirement (Adams and Rau, 2011) is partly answered by the greater existential question, “When will I die?” These findings correspond to longitudinal research indicating that relatively younger retirees, who, ipso facto, expect more years of life, are more likely to engage in bridge employment (Wang et al., 2008).

Feeling tired of work predicted pre-retirees’ and retirees’ preference for not continuing work, paid or voluntary, and, although a pro-active behavioral style and feeling overloaded in work predicted pre-retirees intention to undertake paid or voluntary work in retirement, these qualities did not predict retirees’ intentions (Griffin and Hesketh, 2008). These findings are consistent with research that found physical and psychological health, and the quality of retirees’ exit from the workforce (e.g., choice, timing), predicted their adjustment to retirement (Wong and Earl, 2009; Donaldson et al., 2010) and engagement in bridge employment (Wang et al., 2008). Conversely, retirees who experienced involuntary exit from the labor market may experience difficulty in securing bridge employment (Dingemans et al., 2015). Furthermore, retirees who experienced strain in their career job have been found to take bridge employment in a different field (Gobeski and Beehr, 2009).

Donaldson et al. (2010) found that a psychological sense of mastery (Pearlin and Schooler, 1978) was equivalent in magnitude to conditions of exit as a predictor of adjustment to retirement and stronger than health. Thus, retirees who believe they have independence, choice, and control in their lives are better adjusted. Conversely, recent research revealed a disturbing finding that some individuals evinced the stark contrast in their beliefs that they would never be able to retire out as act of choice because of chronic illness and earning a low income (Schofield et al., 2013). Individuals in this situation return to work as a matter of survival rather than choice and control.

Models of retirees working after retirement (e.g., Beehr and Bennett, 2015) are consistent with the latent and manifest benefits (LAMB) of employment perspective (Jahoda, 1982) that focuses on the social and psychological benefits of employment (e.g., friendship, routine). Financial security, missing the social aspects of the workforce, or a wish to upgrade skills are reasons given for returning to work (Schlosser et al., 2012). Social contact and friendships provide a conducive context for retirees to consider returning to work post-retirement (Wöhrmann et al., 2013) and this is evident in findings of retirees experiencing aging as social loss (i.e., feeling loneliness) or growth (i.e., continuing to make plans) who were, respectively, 1.8 and 2.10 times more likely to return to work (Fasbender et al., 2014). Conversely, experiences of aging as learning more about oneself reduced the chances of employment (Fasbender et al., 2014). Notwithstanding the potential psychosocial benefits (Wang and Shi, 2014), it should be noted that there is evidence of psychosocial harm in the form of discrimination against older workers (Hippel, 2011; Adair et al., 2013).

The social and economic context of the current research is a response to alarming reports into the aging Australian population (e.g., Productivity Commission, 2005; Department of the Treasury, 2010; Actuaries Institute, 2012). So pressing is the need to retain individuals in the labor force that the Australian Government Intergenerational Report (Department of the Treasury, 2010) called for removal of barriers to workforce participation as a requirement to encourage the growing mature age population to remain within or re-join the labor market. Initiatives recommended by the Australian Government (Department of the Treasury, 2010) and the Productivity Commission (2005) aim to retain individuals in the workforce longer (e.g., raising the qualifying age for retirement). Beyond material incentives and disincentives recommended in these reports, there is a need to identify the psychological factors that motivate or de-motivate a retiree to return to paid or unpaid work. The most recent research by the Australian Bureau of Statistics [ABS] (2013a) found that in individuals 45 years and over who had retired and returned to work did so because of financial need or as a way of combating boredom.

Career adaptability in older workers not yet retired is positively associated with job satisfaction; however, the effect is significantly stronger in relatively younger workers (Zacher and Griffin, 2015). Furthermore, older workers’ motivation to continue working did not moderate the relationship between career adaptability and job satisfaction (Zacher and Griffin, 2015). For the current research, we aimed to explore the psychological reasons and benefits for retirees returning to work, viewed from the perspective of the CCT (Savickas, 2005, 2013). In particular, we sought to explore how retirees expressed their career adaptability with respect to the directionality of its effects. That is, we aimed to explore whether career adaptability is associated with returning to work as a potentiating factor for returning to work, or as an outcome benefit of returning to work. Thus, career adaptability is yet to be explored among retirees and the current study is the first to qualitatively explore retirees’ expression of career adaptability

## Materials and Methods

### Epistemological Assumptions

This research project was formulated on the basis of the post-positivist paradigm (Morrow, 2005). Accordingly, we proceeded on the basis that each participant’s narrative is an idiographic assessment, yet psychological experiences may be discerned from the data and interpreted as themes that subsume meanings common to the participants as a group.

### Researcher-as-Instrument Statement

Again, following Morrow’s (2005) recommendations for conducting and reporting qualitative research, here we present a précis of our characteristics as researchers so that the reader may better understand our different perspectives and potential biases. All three researchers have postgraduate degrees; thus, creating a research team comprising a transdisciplinary admixture of adult education, career development, and psychology. The first two authors have worked in aged-care settings, retirement, and mental health, respectively. There were meetings between the first and second authors to discuss data collection and analysis, and, similarly, there were regular meetings between the second and third author to discuss the same, except with the third author acting as an objective auditor separated from data collection and analysis. The university’s Human Research Ethics Committee approved the research.

### Participants

The participants were 22 retirees, 13 male and 9 female, whose ages ranged from 56 to 78 years (*M* = 68.24 years, *SD* = 6.41, *Mdn* = 69). Participants were invited to participate by use of a promotional message distributed via the social network of retirees who had an association with the aged care service at which the first author worked on a part-time basis. The key recruitment criteria stipulated that each participant be self-identified as a retiree who had retired up to 1 year prior to the study (*M* = 6.72 years, *SD* = 4.17, *Mdn* = 6.25), and currently engaged in some form of paid or unpaid (i.e., “volunteer”) employment. There were no recruitment conditions set on the type of work completed during the participants’ lifetime. Participants reported a mix of high-skill and semi-skilled occupations. Their work included higher-level corporate management occupations in Industrial Relations, Finance, Information Technology and Manufacturing, as well as school teachers, a church minister, an academic professor, administrators, a TV/radio technician, agricultural workers, and a small business person. Just one participant had solely worked along one career path throughout pre-retirement working life as a high school teacher before becoming a Department Head. All other participants reported a mixture of different work throughout their careers. Nonetheless, there was at least one participant in each of the major industry categories recognized by the Australian Bureau of Statistics [ABS] (2013b).

### Procedure

Data collection comprised individual face-to-face semi-structured interviews, with a maximum time limit of 1 h per interview. Each interview started with the researcher asking the participant to describe their career history from their first job through to the time of retirement. Interviews were conducted within a participant’s home or a location they nominated and agreed upon with the researcher. Interviews were recorded and transcribed for later data coding and analysis. To enhance the trustworthiness of the data and its subsequent interpretation, the interviews were conducted in two phases. The first author conducted 17 interviews in the first phase of the research. Following the initial data analysis, described below, the second author conducted another five interviews to ascertain if similar results would be drawn from different interviews by a different interviewer. This audit procedure (Yardley, 2007) was conducted to ensure the credibility and dependability (Morrow, 2005) of the data and data analysis.

During the course of the interviews, participants were encouraged by the interviewer to discuss their employment history, reasons for their decision to “un-retire”, career adaptabilities, and their retirement adjustment and planning. These interview topics were further broken down into additional subtopics that were asked when opportunities for further exploration emerged in the conversation between a participant and the researcher. The majority of participants did elaborate on many of these additional questions that covered subtopics such as their feelings toward working with younger colleagues, their perceived idea of what the younger generation felt toward them, their social support base and their personal level of control over their current life situation. Overall, each participant actively involved themselves in their individual interview with the researcher and spoke about their past career history and their current experiences as a retiree who returned to either paid employment or volunteer work.

### Data Analysis

Thematic Analysis (Braun and Clarke, 2006, 2013) was applied to the qualitative data collected from participant interviews and this allowed transitions in interpretations from a broad reading of the data to a more focused discovery of patterns and developing themes.

#### First stage: Data Familiarization

The first stage of the thematic analysis involved the researcher transcribing each of the digitally recorded interviews, then reading and re-reading through the transcripts to ensure accuracy and to also become familiar with and immersed within the data.

#### Second Stage: Generating Initial Codes

**FIGURE 1:**
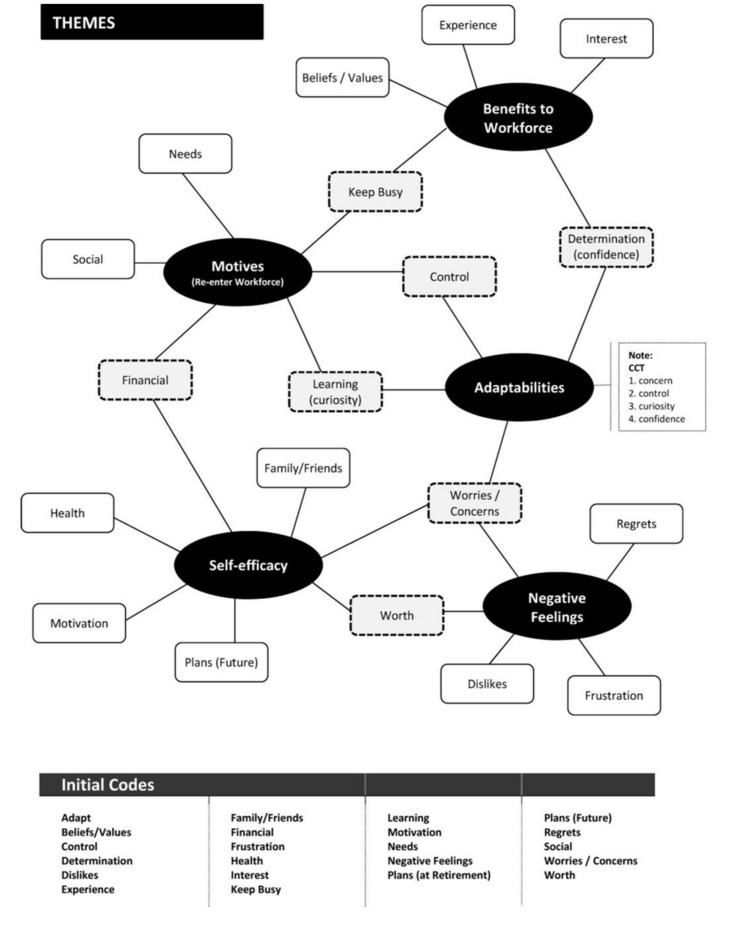
**Map of themes derived from initial codes**.

Developing and assigning coding categories that represented identified topics and themes were then completed during the second stage. Each interview transcript was re-read with initial codes added to any word, sentence or paragraph that the researcher considered as noteworthy to the overall analysis and relevant to the research scope. The initial codes are shown in **Figure 1.**

#### Third Stage: Searching for Themes

As a third stage, the coded data identified from the interview transcripts were gathered together and then the codes were organized into groupings of higher level themes. Braun and Clarke (2006) suggest the development of a thematic map or network to aid the generation of themes. While conducting this form of analysis, the interviewer produced a thematic map shown in **Figure 1**, which assisted in identifying emerging themes by linking and grouping initial codes into overall themes.

#### Fourth Stage: Reviewing Themes

This stage involved checking the themes against the interview transcripts to determine that they told a convincing story of the data and answered the research question. A refinement of the thematic map highlighted that a number of initial codes were now being shared with more than one theme, which illustrated to the researcher that relationships were forming between themes. To bolster the study’s credibility and dependability, the second author frequently reviewed the first author’s interviews, reactions to interviews, and formulation of results, and also engaged in co-analysis of results with respect to theory. Furthermore, the second author conducted a set of independent interviews to ascertain the credibility and dependability of the findings. Confirmability was addressed by detailed reporting of results, inclusive of verbatim statements, and revelation of researchers’ biases.

#### Fifth Stage: Defining and Naming Themes

This stage involved developing a detailed analysis of each theme as well as working out the scope, focus and story of each. The final naming of each theme was completed with consideration made to names that would convey an immediate understanding of a theme’s reasoning and relevance to the research scope. At this stage, the analysis process became recursive as the researcher ensured the final themes selected were integral to the overall research goal. Theses five stages were reiterated for the data collected in the second phase (i.e., the audit). Thematic analysis of the second set of interviews was conducted in the same way as the first set. Saturation was quickly evident in the same themes being extracted from the secondary interviews.

#### Sixth Stage: Producing the Report

This final stage involved weaving together the analytic narrative and data extracts, and contextualizing the analysis in relation to existing literature. This involved selecting quotes from the interview transcripts that were poignant and most represent the research findings. By using thematic analysis, the researcher was able to link the various concepts and opinions of the participants and compare these with the data that have been gathered from the literature review.

## Results

Twenty-two initial codes were identified during the first stage of the thematic analysis of the interview transcripts. Five key themes were identified on collation of the initial codes of the data: career adaptability resources, reasons to disengage (i.e., retire), reasons to re-engage (i.e., rehire), family, and benefits to workforce. There were aspects of the participants’ interview comments that caused many initial codes to overlap across more than one of these five themes. A mind-map depiction of the themes is presented as **Figure 1.**

Here, the results are reported in terms of the career adaptability resources posited in the CCT (i.e., concern, control, curiosity, and confidence). To conceptually enrich the interpretation, the results are reported in terms of the PWF with respect to work fulfilling needs of survival and power, social connection, and self-determination (i.e., relatedness, autonomy, competence).

### Career Adaptabilities

Concern for the future, sense of control, curiosity of opportunities, and confidence in adapting to future occupations are four career adaptabilities (Savickas, 2013) and each appeared within the interviews when the researcher asked participants to describe their current post-retirement employment situation and future plans. Participant #009 was particularly clear on the theme of adapting by stating that “you adapt or you don’t survive”. Considering retirement as a career transition for each of the participants who returned to paid employment, participants such as #003 stated reasons such as “social interactivity keeping you healthy and active” while participant #005 believed that “contributing to the workforce is about self-worth”.

#### Concern

Keeping busy was a strong need that ran through many of the interviews, making it one of the first initial codes used by the researcher to categorize transcripts. Over half of the participants admitted to ‘semi-retiring’ in that they did retire from their full time employment but immediately went looking for work again as they didn’t want to stop. Three of these participants with ages ranging between 61 and 70 are now working full time hours as high school teachers and a quote from one of them (participant #016) sums up the sentiment they all expressed regarding the reason to keep working. “Unless you’re really committed to retiring and retire. I don’t know if people really want to retire when they do”.

Regarding concerns for the future, participant #005 who is a recently retired high school teacher, expressed worry that her entry into retirement would stop opportunities for her to “grow as a person” both professionally and socially. This fear has made the participant more determined to build on her confidence in adapting to future opportunities or occupations. Participant #016 who is a retiree that returned to employment as a high school teacher noted that many of the younger teachers he works with “are insular and think they know it all”. Instead of arguing against this attitude and showing concern, the participant decided to adapt to the situation and understand how better to approach his teaching colleagues as peers.

Future plans were addressed to participants to gauge how strong their motivation was in regards to planning future use of their skills and experience. Participants such as #011 spoke of having “simplified the goals section” of their life in retirement and would rather react to a situation as it occurs. With a third of the participants identifying previous work within a Christian ministry, participants such as #013 stated that his faith would lead him in future decisions and “will not worry himself into an early grave as he has seen others do”.

#### Curiosity

The curiosity of opportunities was strongly shown by many of the participants in their spoken desires to keep learning new skills. The retired teachers or retirees who returned to teaching were the most vocal during their interviews when the question of further learning opportunities was broached. Participant #009 was open to learning further skills in teaching but not “go out of my way to do something that will be a waste on me”. He made this statement when explaining that he believed additional skills training was better suited to his younger colleagues as he’d already been through this same training over and over again.

Computer skills in particular were highlighted as a necessity with participant #005 believing “you must be computer literate” as it “keeps you in good stead with work related researching” and participant #001 acknowledged that though he is concerned that he is not up to date on computer skills, he understands it is a necessity for him to learn as part of his role as a teacher. Informal learning by gathering skills and knowledge via working with colleagues was also highlighted by a number of participants with participant #010 interested in learning new skills but preferably in a work environment where she can actively learn from other workmates instead of attending a course. This participant has learnt from younger workmates and reciprocated by passing on her experience in a “give and take” gesture. Participant #005 also acknowledged that she enjoys working with younger colleagues in her post-retirement sales role as they have various experiences in the workplace and she “likes to learn from them”. Participant #014 saw retirement as being a chance to try different things and do what he wants to do. This focus leads into the fourth adaptability of control.

#### Control

All participants were asked how much control they felt they had over their retirement lives, with responses ranging from control over finances, time flexibility to family situations. The majority of participants responded that they felt they had a fair amount of control over the course their retirement path followed, though there were a couple of participants who did not. In particular, participant #010 stated that at her current age there were a lot of outside influences such as family that gave her a lot of responsibility but not really any sense of control. Similar to the first explored theme of Motives (to re-enter Workforce), there were a large number of responses that referred to the reasons for re-employment being due to having control over personal timetables and enjoying a flexibility not experienced pre-retirement.

Participant #011 enjoys having the flexibility to work to the hours he wants now with no pressing obligations while participant #012 initially retired due to health issues but decided to only semi-retire from a high level corporate career. Moving into consulting work gave him the freedom to still work in his area of expertise but not have the stress of tight schedules and heavy responsibilities that were experienced beforehand. Participant #011 also spoke of the necessity to adapt to his post-retirement employment due to financial reasons but added that he still wanted control because “when you get past your 60’s, there are private entitlements that you’re entitled to and mine is to keep my privacy and basically run my life as I want to at this particular time.” This statement also highlights the beliefs and values of this participant and leads into the last theme explored for this research, that is, how retirees can be a benefit to the workforce.

#### Confidence

Confidence was explored in depth with each of the interview participants through questioning that focused on their personal belief in their abilities, how it motivated them to return to employment, and also how they persevere in any setbacks to their post-retirement employment and goals.

Participants expressed how they perceived the younger generation would view them as work colleagues. Responses focused on a concern that there would be an ageism issue directed at them by these colleagues. Participant #014, a retired teacher, personally believed that younger workers want things done quickly, which is not how he acts now as a retiree. He believed he would clash with the younger generation if he had to work with them because of this reason. Participant #002 related her fear of having younger colleagues believe she was only working again due to financial hardship and that she would be seen as “elderly”. Participant #004 admitted that after retirement from his work as a pastor he had investigated out of interest, whether it was viable to return to his first career path within the electrical industry. When advised that he would have to undergo further training he decided that being a “white haired apprentice” turned him away from the idea. This same participant though also spoke of his sense of worth and of feeling comfortable in being himself when saying he “Doesn’t believe in trying to act young when you’re not. Comes across as artificial”.

The worth of their skills and abilities in the workforce was explored. Participant #005, though very confident in her abilities, admitted that 12 months after retirement, her confidence level is probably lower and that others would judge her ability if she returned to classroom teaching. Similar to this statement, participant #013 also has faith in his skills and abilities but believed that if he returned to his first career as a mechanic, he would not be quick enough to pick up the needed updated skills and that it would be “too stressful and time consuming”. Past experience tainted the thoughts of another participant who believed that Australia had the attitude of not being interested in employing a person in their 60’s. This experience led the participant to work in the UK for a number of years after retirement, where he felt himself and his skills were more accepted. Another interesting comment was made by participant #010 in saying that with all the various jobs she has completed either paid or voluntary over the years, she did not realize that these could all be collated into her résumé or curriculum vitae. This admission, illustrated that she did not recognize the amount of skills she already possessed and of which would be of interest to an employer. Besides a personal non-recognition of skills, a fear of being seen as lesser than others was also highlighted by a considerable number of participants with the following statement particularly descriptive in explaining the underlying worry. “If they hit me with technical questions, I would have a great deal of trouble and they would look at me and think I was nothing” (participant #015).

When focusing on the motivations to succeed in their post-retirement work and how they keep a self-belief in their abilities, there was a range of responses from participants. Participant #014 expressed his excitement for the volunteer and paid employment work he does, as it gives him a feeling that his skills and abilities are contributing to the community in a positive and helpful way. Participant #001 who had previously explained his frustration at being “overlooked” by colleagues, interestingly provided an explanation to how he does maintain his self-belief in his abilities. “People still keep expecting me to fall off the perch, not literally as in dying but as in retiring. I have no intention of doing that”. This participant felt that everyone just expected him to give up and go away into retirement. His frustration in this constant attitude toward himself (whether perceived or not) makes him more determined to stay and show everyone how wrong they are about him, as he knows he has the ability and skill to succeed. This determined attitude or stubbornness fed his confidence.

### Psychology of Working Framework

With the primary aim of discerning evidence of career adaptability now complete we turn to the paradigmatic PWF with the aim of revealing evidence of working as a means of fulfilling psychological needs.

#### Survival and Power

Consistent with Australian Bureau of Statistics [ABS] (2013a) research that found financial reasons for returning to work in Australian retirees over 45 years of age, the current research affirmed financial exigencies in this much older cohort. Though a number of participants stated that they received a minimal pension or had a small “nest egg” they depended on (i.e., savings, superannuation), it was of interest to find that only six of the twenty-two retiree participants clearly identified financial concerns as a motive for re-seeking employment. Participant #003 spoke of “still working to provide financially… to keep control” while participant #002 was concerned about a dwindling nest egg and not being eligible for full government assistance due to assets. Participant #003 explained further that he did not wish to continue working but it was required at this time to fulfill the retirement plans both he and his wife had currently on hold. At the other end of the scale in regards to financial motives there was one participant who identified time flexibility over money as the main motivation while another stated that “financial need not be the important issue” and is happy to live a life of small expenses and happier for it.

The National Seniors Productive Ageing Centre surveyed mature age Australians about their experiences and perceptions of age discrimination in the labor market (Adair et al., 2013) with the fear of age discrimination and age stereotypes being highlighted as the main perceived threats. Hippel (2011) argued that a person does not necessarily have to believe a stereotype is personally true for it to create a threat to their self-belief or self-efficacy. With this in mind and with reference to the research question asking to what extent friends, family, colleagues, and mass media influence a retired person’s perceived capabilities and interest in returning to work, the research identified where real or perceived threats became apparent in many of the participants’ interview comments. With the initial codes of Dislikes, Frustration, Regrets, Worth and Worries/Concerns being associated with these comments, they were collated under the overall theme of Negative Feelings.

#### Social Connection and Relatedness

When participants were asked to what extent family and friends influenced their confidence and decision making, there was a strong leaning toward the attitude of “putting the family before yourself” as participant #010 explained. As a single parent, participant #011 spoke of how he took a large pay cut and left a high-end corporate life for a career that followed a more flexible path that accommodated his family commitments. This path followed him until retirement and now he works very flexible hours as a handyman so as to be available for his now adult children when needed. Similarly, participant #006 also explained both her and her husband made decisions on where to live in retirement based on the needs of their adult children. This line of thinking ran through a number of participant interviews, highlighting the strong family commitment many retirees factor into their living situation, which of course would also impact on what type of post-retirement employment they aimed for. Children were not the only family members that retirees considered in their retirement plans though. Participant #009 plans to soon look after his wife’s parents in a shared living arrangement, while participant #013 explained that his initial responsibility when he retired was to care for his elderly widowed father. These examples of family commitment illustrate that family, no matter what generation can impact greatly on the decisions of retirees and can impact on their motivation and decisions when looking at re-employment options.

Many of the participants also spoke of the social aspect of their work and acknowledged it as something they missed initially at retirement and became a strong motivator for them to stay active. Participant #008 did not miss the pressure or responsibilities of her previous management job but did miss the public customer service side of her job immensely. Within a few days of retiring this participant was actively looking for volunteer work and found a club that she could become a part of. A number of years later, this participant is still actively involved in the management side of the club and appreciates the constant support from her social and volunteer circles. Participant #011 spoke about self-responsibility and referred to the social aspect as most important in providing motivation to keep working as well as being available to provide advice if required. Similar in providing advice, participant #004 hopes to open up communication and foster a grandparent/grandchild relationship with the younger generation that he ministers too in a voluntary capacity. In each of these situations, the participant has seen the social element outweigh any other motive to continue working into retirement.

#### Autonomy

Frustration with their current situation was a strong feeling that surfaced in a small number of the interviews, particularly amongst the male retirees interviewed. Financial, as mentioned under the Motivation theme was one cause of frustration but a more insidious issue was that of not feeling their worth and their past experience not being acknowledged. As a highly qualified and experienced teacher, participant #001 described in detail how he felt in his post-retirement teaching role at a school by stating that “I’m sort of stuck in the predicament where I’m enjoying what I’m doing here but only half my skills are being tapped into”. Feeling there is no way to fix this issue of being overlooked, this participant expressed how “fed up” he was in doing courses and burning himself out, with no promotional outcomes. Similarly, participant #009 strongly believes he was considered an “old grump” at his final school, which caused him to make the drastic decision to retire out of anger. This issue was linked back by the participant during the interview to a sense of frustration he felt at the time within the school and one he regretted following into the path of retirement. In both accounts, these participants highlighted the dislikes, regrets and frustrations they experienced as older workers and how their personal feelings of worth were shaken due to how they felt their colleagues treated them. “Initially retired due to not feeling appreciated and validated” (participant #009). Self-responsibility with a job that gave a flexible timetable attracted participant #011 to continue with some form of employment after retirement. Both of these examples highlighted personal for autonomy over financial needs as the motivation to work post-retirement.

Six of the participants worked solely as volunteers with no financial incentive and with none of them identifying financial concerns. A participant who volunteers his time as a retired pastor stated that the small pension he does receive is seen “as a bonus” and not what he relies on solely. Instead of the financial benefits, it was more about the idea of control being the motivation and he saw retirement as the chance for leaving behind the feeling of compulsion and now allowing himself to be involved “when he wants and because he decided to”. Many of the participants expressed the desire of selecting work in retirement that gave them the flexibility or control over when and where they worked with participant #011 best describing the decision as being able to enjoy work that is now “more comfortable and you don’t have people breathing down the back of your neck”.

#### Competence

With a father “who grew up in the Depression and made do”, participant #013 explained his attitude toward always recycling and reusing resources being the link to his feeling of still wanting to be active in retirement and reuse the abilities he has. Participant #012 who is an industrial chemist and engineer that moved into senior management then consulting roles stated that from experience he believed that anyone in consulting work had to have a very clear understanding of their industry or “they won’t be asked again”. Initially after retiring he was “headhunted” by corporations for his technical expertise that even after 25 years he still found “was fresh” in regards to his skills and knowledge. Participant #007 had a similar experience post-retirement in that his previous high level management work in Information Technology and the Finance industry made him a sought after trouble-shooter consultant for corporations and government agencies. In both these examples, the participants’ experience and knowledge was still relevant to their respective industries and an asset to the following generation. Participant #013 also follows these examples by stating that he “sees the younger generation bringing the enthusiasm and technology… and he brings experience and knowledge”.

Transferring previous skills into a new career path post-retirement was also highlighted by a selection of participants. In particular, participant #005 now works post-retirement as a part-time salesperson for a well-known kitchen appliance brand but previously to this her work as a high school teacher focused on home economics and catering subjects. During the interview with the researcher, the participant acknowledged that she was carrying on her teaching specialty into her new sales work without having realized it. Again, this example shows how industry skills and experience are still relevant into retirement and sought after by the workforce.

While speaking of their career histories, all participants whether they were aware or not, automatically included an explanation of their work ethic with responses including terms such as “self-worth”, “staying focused”, “responsible”, “you adapt” and “good communication”. Participant #013 mentioned that his early career life in the military prepared him “to make sacrifices and not be selfish” which he sees as an advantage he has over the younger generation. The majority of participants spoke of experience being a major advantage they had in regards to their previous careers and many have been involved in and seen “huge changes in technology”. Communication and people skills were also mentioned as highly valuable by a number of participants when noting their worry about the younger generations. Participant #010 highlighted that younger colleagues she worked with “did not know how to approach people” and she believed she could teach them by example. All of these competencies mentioned by participants are writ large in the literature on employability as being in demand in the workforce (Rothwell, 2015).

## Discussion

This research discerned a group of retirees’ reasons to re-engage in working, to seek “rehirement”. The motives for a retiree to re-enter the workforce, the negative feeling they experienced during this life transition, the self-efficacy issues they now experience, how they adapt to this new stage of their life and finally what benefits they bring to the workforce. The research found participants expressed the need to be re-hired was about keeping busy and wanting to experience a feeling of self-worth. For these research participants, a post-retirement job represented a chance to contribute to society, interact with co-workers and/or keep their minds and bodies active. In this way, the current study found results that are consistent with the argument that working meets psychological needs, as posited in the PWF (Blustein, 2006, 2013). As for the primary objective, this study reveals the presence of the four career adaptability resources with retirees.

### Methodological Trustworthiness and Limitations

This qualitative study used thematic analysis (Braun and Clarke, 2006) to investigate career adaptability in a bounded sample of retirees who had returned to work. The design and analytic protocol of this study ensures trustworthiness with respect to the criteria of sound qualitative research according to Morrow (2005). For the sake of establishing credibility of the results, this study followed the published and established protocol for thematic analysis (Braun and Clarke, 2006). The iterative process of discussions between the three authors (i.e., 1 with 2, and 2 with 3) during the coding and development of themes ensured that the findings were credible and dependable. This iterative process operationalized an audit, following the recommendations by Yardley (2007).

With respect to transferability of the findings, it is important to note that the participants were relatively well resourced with respect to capital—financial, social, and psychological. Therefore, the results of this study should not be taken as transferable to retirees who lack such capital. Thus, the results may be used to inform considerations of the experiences of retirees who return to work several years after their separation from the full-time workforce and who enjoy a relatively secure financial and social status.

### Implications for Theory and Research

Exploring retirement from the perspective CCT (Savickas, 2005, 2013) is novel, if not ironic, because career theories are predominantly focused on career in the traditional sense (i.e., a person’s working life) and not so much retirement, which is the transition representative of the putative final stage in a career, namely, disengagement/decline (Super, 1980). The findings of this research add to current body of empirical research that pertains to the notion of career adaptability in the CCT (Savickas, 2013). The participants expressed concern with respect to their future; they expressed control with respect to keeping active and engaged; they expressed curiosity with respect to learning new skills and knowledge; and, they expressed confidence in knowing that they could make a positive contribution. Thus, the current study provides evidence that career adaptability is not only a psychological dimension of emerging adults and mid-career adults in the workplace. This research indicates that career adaptability may be a life-long psychological resource for all people interested in working. Finding manifestations of career adaptability in retirees demonstrates its potential as a developmental theory (Savickas, 2002) that conceptually challenges current conceptualizations of the stage disengagement/decline.

The Career Adapt-Abilities Scale (CAAS; Savickas and Porfeli, 2012) has been designed specifically to align with career adaptability as defined in the CCT. However, extant research into career adaptability and its measurement is focused predominantly on youth and relatively younger adults, and not retirees. Given this limitation, the findings of this study are reason to explore the psychometric properties of the CAAS in older adults, both those in the transition of retirement and those returning to work after retiring. Such research may provide evidence of convergence with other psychometric measures that tap psychological dimensions of retirement, such as the Retirement Resources Inventory (Leung and Earl, 2012) or the Transition to Retirement Questionnaire (Maggiori et al., 2014). For example, measures of social support in the Resources Retirement Inventory may moderate a relationship between Career Adapt-Ability and engagement in bridge employment. Alternatively, there may be different levels of Career Adapt-Ability within the different types of retirees (e.g., Adventurers, Retreaters) differentiated by the Transition to Retirement Questionnaire.

Career adaptability can be conceptually associated with the notion of employability (Fugate et al., 2004; Rothwell, 2015) and intrapreneurship (Di Fabio, 2014). Although a retiree may want to decent work, his or her desire may not correspond with his or her employability in the labor market at any point in time, particularly given issues of age-related biases (Guilbert et al., 2015). Thus, it is appropriate to investigate if the factors that constitute young workers’ employability are similar to the factors for older workers. Accordingly, exploring the measurement properties of scales of employability (e.g., Fugate and Kinicki, 2008; Rothwell et al., 2008, 2009) across different age groups may bear conceptual and pragmatic fruits.

Senescence is associated with changes in cognitive performance, which has been highlighted by PWF scholars as an issue of concern for retaining older workers in the labor market (Sterns and Sterns, 2013). Although, there is some debate about the conceptualization and measurement of emotional intelligence (Mayer et al., 2008), there is an emerging body of research that reveals relations between academic cognitive performance and emotional intelligence (Qualter et al., 2012; Perera, 2015). Given the association between career adaptability and emotional intelligence (Coetzee and Harry, 2014) it is tempting to speculate that emotional intelligence may have a compensatory effect on cognitive decline older workers’. Indeed, there is a promising program of vocational psychology research that demonstrates that emotional intelligence contributes above and beyond cognitive ability to resilience (Di Fabio and Saklofske, 2014; Di Fabio, 2015) and career decision-making (Di Fabio and Kenny, 2011; Di Fabio et al., 2013; Di Fabio and Palazzeschi, 2014) in younger people. Thus, there is scope to explore how best to utilize older workers’ emotional intelligence to enhance their employability in association with resilience and career decision-making.

### Implications for Practice and Policy

Employability is of central concern to career development practitioners and policy makers (Guilbert et al., 2015) and there is scope to design programs to assist retiree job seekers to recognize, rebuild, and reuse their expertise to refresh the labor market. Such interventions may be incorporated in retirement planning that takes a psychological perspective, not just a financial perspective (e.g., Donaldson et al., 2010; Adams and Rau, 2011). Career intervention programs could be developed to incorporate relevant constructs, such as career adaptability, so that employment and training services, and financial planning services, can appropriately support retiree job seekers (Earl et al., 2015). Despite the ostensible benefits of planning for quality of life in retirement (Adams and Rau, 2011), some caution is warranted because there is evidence that neither pre-retirement nor post-retirement planning contributes to adjustment (Donaldson et al., 2010).

It is important to note that the developmental needs of younger workers and students are different to the needs of older workers and students. Therefore, PWF scholars have argued that holistic career development and employment services for adult workers should be visible, accessible, and designed for their specific needs (Juntunen and Bailey, 2013). Indeed, there is a lack of specificity evident in the Sustainable Development Goals (United Nations, 2015). The wording of the Goals is sufficient to cover older people with respect to diminishing poverty (i.e., Goal 2) and improving city design (Goal 11); however, Goal 8 (viz., Promote sustained, inclusive, and sustainable economic growth, full, and productive employment and decent work for all) does not specifically address the unique needs of older workers. The wording is directed at young people and people with a disability. Perhaps, as the Goals are progressively implemented by various nations, older workers will be given more of a presence in subsequent revisions of their wording, particularly as governments clamber to fill gaps in the labor market.

## Conclusion

This research shows that it is possible for an aged person to continue to be concerned and curious about work, in control of opportunities and resources, and confident that they may thrive and make an authentic contribution. We suggest that the notion of career adaptability can be an important focus of research and development so as to foster retirees’ healthy re-engagement in the world of work.

## Author Contributions

All authors were involved in conceptualization of the research problem, research design, and interpretation of data. JL and PM collected data from interviews with participants, and HP acted as the auditor of results and interpretation. All authors contributed to the final writing process.

## Conflict of Interest Statement

The authors declare that the research was conducted in the absence of any commercial or financial relationships that could be construed as a potential conflict of interest. The reviewer LP and handling Editor declared their shared affiliation, and the handling Editor states that the process nevertheless met the standards of a fair and objective review.
